# The impact of health information echo chambers on older adults avoidance behavior: the mediating role of information fatigue and the moderating role of trait mindfulness

**DOI:** 10.3389/fpsyg.2024.1412515

**Published:** 2024-08-16

**Authors:** Fuxiu Zhong, Chenyu Gu

**Affiliations:** ^1^School of Journalism and Communication, Minjiang University, Fuzhou, China; ^2^Department of Hepatobiliary Surgery Nursing, Fujian Medical University Union Hospital, Fuzhou, China; ^3^Fujian Digital Media Economy Research Center, Fujian Social Science Research Base, Fuzhou, China

**Keywords:** echo chambers, older adults, health information avoidance, trait mindfulness, information fatigue, SSO framework

## Abstract

**Background:**

In the digital media era, the prevalence of algorithm-driven content push has intensified the health information echo chambers phenomenon, characterized by excessive homogenization and overload of information. This phenomenon may negatively impact the older adults, a “digitally vulnerable” group, by limiting their access to diverse health information and potentially inducing health information avoidance behaviors. However, the psychological mechanisms within this impact process remain unclear, and this study aims to explore them. Additionally, current study introduces trait mindfulness as a potential intervention tool in reducing health information avoidance behavior among the older adults.

**Methods:**

This study constructs an impact model based on the Stress-Coping Theory and the Stress–Strain-Outcome (SSO) framework. It identifies health information similarity, relevance, and overload as characteristics of the echo chamber, constituting the Stress part; information fatigue as the Strain; and health information avoidance as the Outcome, with trait mindfulness serving as a moderating factor between Strain and Outcome. A cross-sectional survey of 236 older adults individuals aged 60 and above was conducted, and the collected data were analyzed using SmartPLS and SPSS.

**Results:**

The study found that health information similarity and overload significantly increased health information fatigue among the older adults, thereby promoting avoidance behavior, but no significant correlation was found between relevance and fatigue. While there was no significant correlation between trait mindfulness and health information fatigue, it effectively moderated the avoidance behavior induced by fatigue.

**Conclusion:**

Current study reveals the impact of the health information echo chamber phenomenon on health information avoidance behavior among the older adults and the moderating role of trait mindfulness. It emphasizes the importance of breaking the health information echo chamber and promoting diverse information dissemination to improve the health information behavior of the older adults. Furthermore, the moderating role of trait mindfulness demonstrates positive intervention potential, providing the older adults with a psychological adjustment mechanism to accept and process health information with a more open attitude, thereby reducing unproductive avoidance behavior.

## Introduction

With the development of digital technology and the internet, the communication and accessibility of health information have surpassed any prior point in history. Yet, many individuals still choose to avoid online health information, not only placing themselves at imminent health risks but also posing challenges to public health (Soroya et al., 2021). The negative impacts of health information avoidance are particularly severe for the older adults due to the decline in physiological functions, which may lead to more health issues. Additionally, their health literacy generally falls below that of younger people who have grown up in a digital environment. During the COVID-19 pandemic, digital technology was widely adopted and rapidly popularized, leading to a more frequent use of digital technology among a large number of older adults people. This has made the intertwining and conflict between digitalization and aging an increasingly significant issue in social management. Today, the internet has become an important channel for the older adults to access health information. However, faced with a vast amount of online health information of varying quality, more and more older adults individuals are beginning to choose avoidance (Zhao et al., 2022). This may result in a reduction in participation in health activities and health monitoring behaviors, or even missing opportunities for disease diagnosis and treatment, thereby placing them at health risk (Chae, 2015). Against the backdrop of a global trend toward population aging, the behavior of health information avoidance among the older adults has become an important research topic in the fields of public health and information management.

The current online information environment is largely shaped by data-driven algorithms, resulting in a more severe echo chamber effect than seen in traditional media environments. This significantly impacts users’ health behavior patterns (Cinelli et al., 2021). The older adults have a particularly urgent need for online health information; digital platforms offer them a vital source of health information, aiding in personal health management, understanding of illnesses, and treatment options. However, the echo chambers created by algorithms also limit the diversity of health information that the older adults are exposed to. When older adults repeatedly encounter health information pushed by algorithms, it may lead to health information avoidance behaviors (Jiang, 2022). Yet, there is a scarcity of research exploring the impact of health information echo chambers on the older adults health information avoidance behaviors and the mechanisms for intervention.

To address the gaps in existing research, current study, based on the stress coping theory and Stress–Strain-Outcome (SSO) framework, constructs a model to explore the impact of online echo chamber information characteristics (health information similarity, relevance, and overload) on health information avoidance among the older adults. It also examines the mediating role of trait mindfulness in this process. This cross-sectional survey distributed questionnaires to 236 older adults individuals. The findings offer theoretical foundations and practical guidance for creating a more equitable, inclusive, and age-friendly health information environment.

## Theoretical framework

### Older adults health information avoidance

Just as information seeking and serendipitous discovery are integral parts of human information behavior, information avoidance reflects a distinct phenomenon where information is deliberately not acquired (Sweeny et al., 2010). Early in the 1940s, psychology scholars regarded the phenomenon of information avoidance as a result of selective exposure to specific stimuli, closely related to the concept of Selective Exposure proposed on the basis of cognitive dissonance theory (Hyman and Sheatsley, 1947). Subsequently, in the 1990s, the information behavior model proposed by Wilson and the comprehensive model of information seeking constructed by Johnson and Meischke established the existence of information avoidance behavior, suggesting that individuals might avoid seeking information due to stress and cognitive factors leading to unmet information needs (Johnson and Meischke, 1993; Wilson, 1999).

In recent years, with the increase in public health service demand and the development of digital communication technology, health information avoidance has become an important direction in the study of information avoidance (Howell et al., 2020). It has been extensively researched in fields such as health psychology, information management, library and information science, and health communication (Khaleel et al., 2020). Research on health information avoidance mainly expands inwardly and outwardly. The inward expansion focuses on individual factors leading to health information avoidance (such as emotions, information processing styles) and factors related to the information itself (Howell and Shepperd, 2017). The outward expansion focuses on the impact of the level of social support, social networks, and the richness of social capital on health information avoidance (Howell and Shepperd, 2013).

Although scholars have made some progress in researching health information avoidance, our literature review reveals that there is still a lack of research on health information avoidance behavior among the older adults. Due to physical decline, the older adults have a more urgent need for health information (Xie et al., 2022). Studying the health information avoidance behavior of the older adults is crucial for optimizing their health management and enhancing the effectiveness of public health strategies, especially in the context of health information, where the older adults face unprecedented opportunities and challenges in accessing information (Nikou et al., 2020). By understanding the reasons and mechanisms behind the older adults avoidance of health information from the perspective of digital information dissemination, we can develop more targeted health education and intervention measures. This, in turn, promotes healthy behaviors among the older adults, reduces health risks caused by information avoidance, and has significant social and public health value, especially in the face of the growing challenges of an aging society.

### The echo chamber effect

The concept of the echo chamber effect was first introduced by Sunstein in the book “Information Utopia: How People Produce Knowledge.” This concept describes a process in which individuals actively filter the information they are exposed to, creating an enclosed information environment similar to an echo chamber (Sunstein, 2006). Within such an environment, individuals repeatedly encounter information they are predisposed to seek, leading to an increase in the homogeneity of information and a narrowing of information exposure, ultimately influencing individual beliefs (Peng and Liu, 2021). In the digital media era, the dissemination and push of information rely on algorithms. Once a user shows a preference for certain types of content, it is likely that they will continue to receive similar content, which aligns closely with the formation path of the echo chamber effect (Piao et al., 2023). Although the concept of the echo chamber effect existed before the rise of algorithmic pushes, the popularity of algorithmic pushes has led more scholars to pay attention to its reinforcing effect on the echo chamber effect (Ross Arguedas et al., 2022). Nowadays, an increasing number of scholars have begun to study the echo chamber effect in the context of digital health information dissemination and have revealed the negative impacts of overly homogeneous health information on individuals (Wang et al., 2022). However, based on a review of the existing literature, we find that few studies have focused on the potential impact of the echo chamber effect on health information avoidance behavior.

The echo chamber effect emphasizes the active selection and narrowing of information exposure by individuals. In the context of health information dissemination, due to the reinforcing effect of algorithmic pushes, the result is the push of homogeneous health information, which may lead to information overload and even exacerbate individuals’ anxiety about their health conditions. Given the physiological decline of the older adults and their relatively lower digital health literacy, they are more likely to be negatively affected by the health information echo chamber effect. Based on this logic, it is necessary for researchers to explore how the health information echo chamber effect influences health information avoidance behavior among the older adults.

### Stress coping theory and the SSO framework

Stress coping theory, originally rooted in psychology, has been widely applied to fields such as information management and user behavior. It aims to clarify how users assess stress situations and demonstrate corresponding cognitive and behavioral efforts (Fang et al., 2011). The theory suggests that individuals follow two parallel paths when confronted with stress: a primary appraisal, including the assessment of the situation’s relevance and threat level, and a secondary appraisal, where individuals evaluate their resources and abilities to manage potential stress situations; these processes occur simultaneously. Coping theory explains concepts such as sources of stress, stress responses, and coping strategies and is extensively used to analyze and interpret individuals’ negative behaviors when faced with stress (Chen et al., 2019).

This theory aligns closely with the Stress–Strain-Outcome (SSO) framework, a comprehensive model for understanding individual behavior, first introduced by Koeske and Koeske (1993). This model posits that external stressors can impose psychological burdens on individuals, leading to negative behavioral responses (Koeske and Koeske, 1993). The efficacy of this model in explaining negative behavioral responses has been widely validated and applied by scholars to research various types of negative online behaviors. For instance, Ma et al. (2022) based on the SSO model, explored the impact of information redundancy from AI recommendation algorithms on users’ discontinuance behavior. Although research employing the SSO model to explore health information avoidance behavior is scarce, health information avoidance behavior exhibits clear negative behavioral traits and falls under a specific category of information avoidance behavior, fitting the SSO model’s application scenario. Furthermore, the negative characteristics of digital health information cocoons, largely stemming from algorithmic push mechanisms (Zuiderveen Borgesius et al., 2016), align with the characteristics of stress sources in the SSO model. Based on the above, this study constructs a framework for the impact of digital echo chambers on health information avoidance behavior among the older adults, grounded in stress coping theory and the SSO framework.

This study identifies three factors—health information similarity, health information relevance, and health information overload—as the sources of stress represented by the echo chamber in the online health information communication. These factors not only shape individual information reception and behavioral responses but also have a direct impact on psychological states. Firstly, one of the main characteristics of the echo chamber effect is the similarity of health information. This similarity leads to individuals being consistently exposed to information that aligns closely with their existing health beliefs. The uniformity of such information can hinder a comprehensive understanding of health risks, thereby increasing uncertainty and anxiety when facing health decisions, and becoming a source of psychological stress (Cinelli et al., 2021). Secondly, in the digital communication environment, information is tailored based on individual interests and past behaviors, meaning it is highly relevant to the individual. While this customization increases the attractiveness of the information, it may also exacerbate biases, leading to a narrow scope in the selection of health information. This over-customization of information flows can restrict opportunities for encountering diverse information, increasing psychological stress when confronted with health issues (Khaleel et al., 2020). Lastly, health information overload is not only a prominent feature of the modern information environment but has also been shown in past research to be a typical manifestation of the information echo chamber. An abundance of constantly updating health information can overwhelm individuals, making it difficult to discern which information is reliable. This flood of information not only consumes cognitive resources but may also cause decision fatigue, adding to psychological stress, especially when making significant health choices (Crook et al., 2016). In summary, this study decides to characterize the echo chamber in the SSO framework through the stressors of health information similarity.

### The impact of health information Echo chambers (stress) on health information fatigue (strain)

The manifestation of echo chambers in a digital media environment is characterized by the repeated exposure to similar information (Cinelli et al., 2021). If the older adults find themselves within a health information echo chamber, the information cocoon are likely interwoven with a certain type of health information, often related to their own health conditions. Given the characteristics of algorithmic information push, it’s conceivable that they might encounter a large volume of push notifications related to such health information (Jennings et al., 2021). Therefore, this study uses health information similarity, relevance, and information overload as characteristics of the health information echo chamber, forming the Stress part of the SSO model.

Health information similarity refers to the consistency or resemblance in content, viewpoint, or structure of the health information that individuals access through digital platforms (Liang and Fu, 2017). This similarity is driven by personalized recommendation algorithms aimed at matching users’ historical preferences and online behaviors. Information fatigue is a state of psychological weariness and declining interest toward information. In the domain of health information, it manifests as a decrease in attention to health information and a negative attitude toward it (Kuem et al., 2021). Based on the Incentive-Sensitization Theory, continuous exposure to similar information may lead to reduced cognitive stimulation and lowered processing motivation because novelty and diversity of information are crucial factors in maintaining cognitive stimulation and interest (Galvao et al., 2013). A high degree of information similarity could lead to mechanical processing of information by individuals, thereby increasing the sense of information fatigue. Hence, this paper proposes the research hypothesis:

*H1a*: Health information similarity positively influences information fatigue.

Health information relevance refers to the degree to which information matches an individual’s health condition, interests, needs, or behaviors. On digital media platforms, algorithms typically recommend “relevant” information based on a user’s past behavior, search history, and preferences (Bode and Vraga, 2018). Although some studies have shown that information relevance can reduce the cost of information search and the effort spent on information processing, long-term frequent exposure to highly relevant information may lead to cognitive and emotional fatigue (Khaleel et al., 2020). This fatigue is not caused by the volume of information but by the lack of sufficient information diversity and depth, leading to monotonous cognitive stimulation and making information processing tedious (Prybutok et al., 2014). Therefore, this paper proposes the research hypothesis:

*H1b*: Health information relevance positively influences health information fatigue.

Health information overload refers to the phenomenon of information overload that occurs when individuals seek, receive, and process health-related information, which is very common in the algorithmically driven media environment (Fu et al., 2020). Information overload can lead to decision-making difficulties, anxiety, and a decrease in information processing efficiency (Crook et al., 2016). With the widespread availability of health information on the Internet, the public can easily access a vast amount of information about diseases, treatment methods, preventive measures, and healthy lifestyles; while this provides convenience for personal health management, it also presents challenges (Khaleel et al., 2020). When faced with a large amount of conflicting, complex, or technical health information, individuals may feel uncertain and anxious, making it difficult to make informed health decisions, especially on digital media platforms driven by algorithms, increasing the risk of encountering information overload (Dong et al., 2024). Previous research has shown that information overload can easily cause individuals to experience information fatigue (Mao et al., 2022), displaying a similar psychological mechanism in the context of health information dissemination. Individuals’ cognitive processing capabilities are limited, and facing a vast amount of health information, individuals must expend significant psychological and cognitive resources to try to understand and filter information, which may quickly deplete their cognitive resources. When cognitive resources are overused, individuals may feel fatigued and unwilling to process more information, leading to information fatigue. Therefore, this paper proposes the research hypothesis:

*H1c*: Health information overload positively influences health information fatigue.

### The impact of health information fatigue (strain) on health information avoidance behavior (outcome)

The concept of fatigue originates from the fields of psychology and medicine, referring to the degree of tiredness perceived by individuals toward certain activities (Aaronson et al., 1999), and has been widely applied to research areas such as information management and news dissemination. This study focuses primarily on information fatigue. Health information fatigue refers to the weariness, decreased interest, or diminished motivation to process large volumes of health information over a prolonged period (Shi et al., 2022). Scholars have conducted extensive research on the causes of information fatigue, such as information overload, the complexity of information, or a mismatch between information and individual needs, all of which can exacerbate feelings of fatigue (Shulman et al., 2021; Sheng et al., 2023). In a state of health information fatigue, individuals may feel overwhelmed by excessive health information, leading to psychological and cognitive discomfort.

health information avoidance behavior refers to the deliberate act of avoiding contact with, searching for, or processing online health-related information, exhibiting a negative resistance to health information (Link et al., 2023). Avoidance behavior may stem from various reasons, including fear of the information content, distrust of the information source, or feeling that the information is irrelevant or too complex to understand (Siebenhaar et al., 2020); its psychological motives can be to avoid cognitive dissonance or to escape fear and anxiety about health risks (Song et al., 2021).

Previous research has demonstrated that information fatigue can trigger negative behaviors (Jia et al., 2023). Compared to younger digital natives, the older adults, due to generally lower health literacy, are more susceptible to the negative effects of online information (He et al., 2020). When the older adults are in a state of health information fatigue, they may find the task of processing these health information both burdensome and oppressive. Over time, this continuous psychological stress and fatigue can gradually diminish their motivation to engage with new online health information (Islam et al., 2020). Based on the protection motivation theory, individuals often use avoidance of stress sources as a self-protection mechanism; therefore, the emotional and cognitive burden brought by online health information may lead to health information avoidance behavior among the older adults to alleviate discomfort and restore psychological balance. Therefore, this paper proposes the research hypotheses:

*H2*: Health information fatigue positively influences health information avoidance behavior.

*H2a*: Health information fatigue mediates the impact of health information similarity on health information avoidance behavior.

*H2b*: Health information fatigue mediates the impact of health information relevance on health information avoidance behavior.

*H2c*: Health information fatigue mediates the impact of health information overload on health information avoidance behavior.

### Trait mindfulness as a potential moderating intervention

The concept of mindfulness originates from meditation practices in Buddhism, referring to the intensity of attention (Jacobs and Blustein, 2008), and is generally divided into state mindfulness and trait mindfulness in research (Randal et al., 2015). Trait mindfulness, as a psychological characteristic, refers to an individual’s capacity to focus on present occurrences with a non-judgmental and accepting attitude toward any thoughts or feelings (Keng et al., 2011). It is considered a stable psychological state requiring conscious focus on specific objects, being present in the moment. Trait mindfulness is thought to effectively mitigate the harm that negative stimuli can cause to an individual’s mental health and reduce the resultant negative behaviors (Brown and Ryan, 2003). To explore sustained rather than transient effects, this study aims to examine the potential of trait mindfulness in intervening in health information avoidance behavior among the older adults.

Previous research indicates that mindfulness can effectively help individuals cope with stress and alleviate the fatigue generated by stress (Daya and Hearn, 2018). When faced with health information that may cause fatigue, trait mindfulness enables individuals to maintain an open mindset, accepting rather than avoiding the discomfort these messages may bring (Esmaeilzadeh, 2020). Through such an accepting attitude, individuals can explore and process health information more proactively instead of avoiding it to escape discomfort. Trait mindfulness also promotes deep reflection and inner processing capabilities when individuals confront information (Gao et al., 2021; Hsieh et al., 2021), helping the older adults not to feel bored or fatigued by the monotony and repetitiveness of similar or related health information, thus directly reducing the generation of fatigue. Additionally, evidence suggests that individuals with high trait mindfulness possess better emotional regulation abilities and mitigate negative behaviors produced by negative emotions (Kriakous et al., 2021; Gu et al., 2022), potentially alleviating the negative impact caused by information fatigue. Individuals with a high level of mindfulness can better manage their responses to health information (Walsh and Mitchell, 2005), not allowing fatigue and discomfort to dictate their information processing methods. This enhanced self-management means that even when faced with a vast amount of health information that could cause fatigue, individuals are likely to maintain a lower tendency to avoid and more actively process and utilize this information.

In summary, an individual’s level of trait mindfulness has the potential to inhibit the generation of health information fatigue. Furthermore, by enhancing individuals’ acceptance, deep reflection, and emotional regulation capabilities, trait mindfulness could effectively reduce health information avoidance behavior caused by fatigue. Individuals are thus more likely to engage with health information in a positive and constructive manner, maintaining good contact and utilization of information even within health information echo chambers. Therefore, this paper proposes the following research hypotheses:

*H3a*: Trait mindfulness negatively impacts health information fatigue among the older adults.

*H3b*: Trait mindfulness negatively moderates the impact of health information fatigue on health information avoidance behavior among the older adults.

### Conceptual model

**Figure 1:**
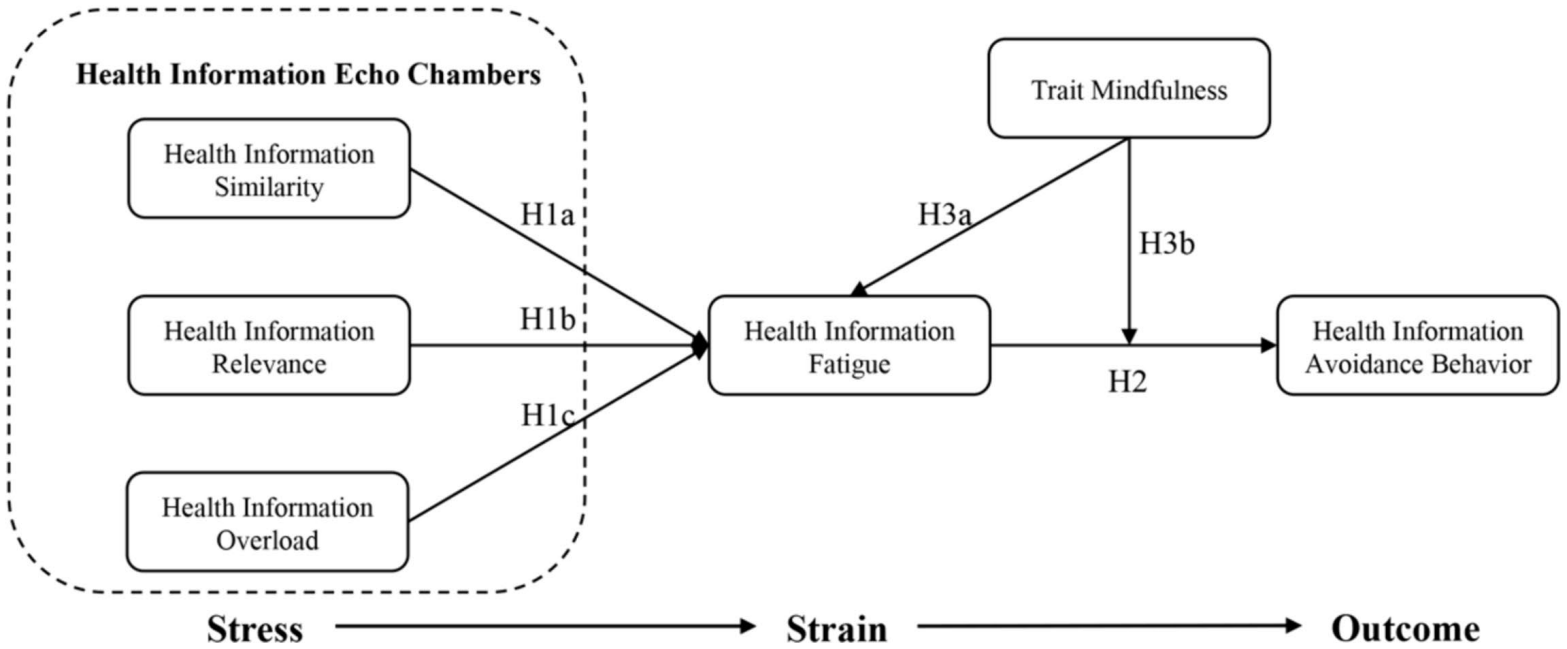
Conceptual model.

Based on the above information, the research model of current study is shown in Figure 1. In the model, we include age, gender, education level, and average daily internet usage time as covariates.

## Materials and methods

Current study is based on the SSO framework, characterizing the Stress part as the digital information echo chamber in terms of health information’s similarity, relevance, and overload; Strain as health information fatigue; and Outcome as health information avoidance among the older adults. The aim is to investigate how the health information echo chamber, driven by algorithmic push, triggers health information avoidance among the older adults through influencing health information fatigue. As this research focuses on the older adults population, it is essential to ensure that our sample consists of individuals aged over 60 years. Our study is a cross-sectional investigation employing a questionnaire survey method. To ensure the clarity of question wording, a pilot test was conducted with a small group of participants. Before participating in the survey, participants were informed of their right to withdraw, confidentiality, and anonymity. Computers, tablets, and mobile phones were all available for completing the survey. After obtaining consent, participants began answering our official questionnaire.

### Participants and procedures

Given that the target sample of this study consists of older adults digital users, who may face certain challenges in recruiting effective samples and understanding the questionnaire, researchers requested the assistance of students from four classes at their university. These students were invited to serve as survey assistants to conduct the questionnaire survey among their older adults family members. Before the survey commenced, the researchers trained these assistants to ensure they could accurately convey the questionnaire items to the older adults respondents. The study initially collected 312 questionnaires, of which 76 were excluded as invalid, resulting in 236 valid questionnaires with a validity rate of 75.64%. The criteria for selecting valid questionnaires included: (1) correct answers to valid screening items in the questions; (2) total answering time exceeding 1 min; and (3) avoidance of selecting the same value for eight consecutive questions. The specific information of the sample is shown in Table 1.

**Table 1:** Statistical table of basic information of effective samples.

Statistical items	Specific content	Statistical value	Percentage
Gender	Male	88	37.3%
	Female	148	62.7%
Age	60 ~ 65	194	82.2%
	66 ~ 70	26	11.0%
	71 ~ 75	15	6.4%
	Over 75	1	0.4%
Educational background	Primary School	75	31.8%
	Middle School	44	18.6%
	High School	63	26.7%
	Undergraduate	50	21.2%
	Master and Doctor	4	1.7%
Social media usage duration	Less than 1 h/day	8	3.4%
	2 ~ 4 h/day	55	23.3%
	4 ~ 6 h/day	97	41.1%
	6 ~ 8 h/day	70	29.7%
	Over 8 h/day	6	2.5%

### Measurements

This study aims to measure 6 latent variables: health information similarity, health information relevance, health information overload, health information fatigue, health information avoidance, and trait mindfulness.

The health information similarity scale, adapted based on the context of health information dissemination, follows the research of Walsh and Mitchell (2005), consisting of four measurement items: (1) I feel that the health information pushed to me online is very similar. (2) The health information I see on different websites is almost the same. (3) Regardless of which website I visit, the health information provided is very similar. (4) Online health information usually repeats the same views and advice.

The health information relevance scale is based on the study by Prybutok et al. (2014), including four measurement items: (1) The health information content pushed to me online mostly relates to me. (2) The majority of online health information is not applicable to me. (3) The majority of online health information is not important to me. (4) Online health information is usually highly relevant to my health needs.

The health information overload scale, derived from the research of Laato et al. (2020), comprises three measurement items: (1) There is too much health information pushed to me online, causing me disturbance. (2) I have seen too much health information online, but most of it is similar. (3) The excessive amount of health information online makes it difficult for me to notice important information.

The health information fatigue scale is adapted from Ahsberg’s study (Ahsberg, 2000), containing three measurement items: (1) Sometimes I feel tired when I see health information pushed online. (2) I have lost interest in health information online. (3) I find it boring to read health information online.

The health information avoidance scale follows the research of Howell and Shepperd (2016), including five measurement items: (1) I would prefer not to receive health information online. (2) I prefer to avoid health information online. (3) When it comes to online health information, ignorance is bliss. (4) I usually do not click on links to health information. (5) I try to minimize my exposure to health information online.

The trait mindfulness scale originates from the study by Turel and Osatuyi (2017), comprising five measurement items: (1) I am easily distracted, I am always distracted when I do something. (2) I have trouble concentrating and paying attention to things I am doing. (3) I find myself doing things without paying attention. (4) It seems I am “running on automatic” without much awareness of what I’m doing. (5) I find it difficult to stay focused just on what’s happening in the present, without making any judgment about it.

All measurement instruments used in this article are sourced from existing mature research. The average value of each sub-measurement item is taken to form the score of each latent variable. To simplify the score selection process for the older adults, a unified Likert 5-point scale is used for measurement. A pre-survey was conducted with 10 samples to ensure all measurement items could be accurately understood by the older adults.

### Data analysis

The model constructed in this study does not derive from existing models. Although it is built on theoretical frameworks and logical reasoning, it remains exploratory in nature. Additionally, due to the challenges associated with recruiting a sufficient number of older adults participants, while the sample size meets the statistical analysis standards, it is not particularly large. Therefore, following the recommendation of Hair et al. (2012), this study employs SmartPLS’s Partial Least Squares Path Modeling (PLS) for testing.

## Results

### Measurement of model

Firstly, the data underwent reliability and validity tests, with the specific results shown in Table 2. The factor loadings ranged between 0.625 and 0.941, indicating that all measurement items met the standards; the Cronbach’s alpha values of each latent variable were all greater than 0.8, and the Composite Reliability (CR) values exceeded the acceptable standard of 0.7, demonstrating that the scales’ reliability was satisfactory (Hair et al., 2019). Additionally, the Average Variance Extracted (AVE) values of all latent variables were greater than 0.5, indicating adequate convergent validity (Fornell and Larcker, 1981). Examination of the Variance Inflation Factor (VIF) values for each factor revealed that all results were below 10, indicating no multicollinearity issues in this study (Hair Jr. et al., 1998).

**Table 2:** Reliability and convergent validity analysis.

Latent variable	Items	Factor loadings	VIF	Cronbach’s α	CR	AVE
Health information similarity	Similarity 1	0.840	1.895	0.858	0.862	0.701
	Similarity 2	0.827	2.030			
	Similarity 3	0.849	2.152			
	Similarity 4	0.833	1.946			
Health information relevance	Relevance 1	0.798	1.754	0.839	0.863	0.670
	Relevance 2	0.824	2.206			
	Relevance 3	0.798	1.834			
	Relevance 4	0.852	1.777			
Health information overload	Overload 1	0.625	1.355	0.863	0.934	0.664
	Overload 2	0.918	1.768			
	Overload 3	0.872	2.008			
Health information fatigue	Fatigue 1	0.931	3.363	0.929	0.931	0.876
	Fatigue 2	0.941	3.913			
	Fatigue 3	0.936	3.654			
Health information avoidance	Avoidance 1	0.830	2.697	0.906	0.908	0.727
	Avoidance 2	0.898	3.836			
	Avoidance 3	0.827	2.146			
	Avoidance 4	0.839	2.262			
	Avoidance 5	0.866	2.600			
Trait mindfulness	Mindfulness 1	0.880	3.261	0.943	0.955	0.814
	Mindfulness 2	0.920	4.713			
	Mindfulness 3	0.906	4.326			
	Mindfulness 4	0.883	3.053			
	Mindfulness 5	0.922	4.107			

Subsequently, discriminant validity was assessed, with results presented in Table 3. The square roots of the Average Variance Extracted (AVE) values (numbers on the diagonal) exceeded the Pearson correlation coefficients between variables, indicating that the discriminant validity of the scales met the standards (Fornell and Larcker, 1981). To further validate the absence of common method bias in the data, this study employed Harman’s single-factor test method. Under unrotated conditions, there were five factors with eigenvalues greater than 1, and the first factor accounted for only 34.418% of the total variance, which did not exceed the standard threshold of 40% (Podsakoff et al., 2003). Based on this analysis, the data in this study were not affected by common method bias.

**Table 3:** Discriminant validity analysis.

	Similarity	Relevance	Overload	Fatigue	Avoidance	Mindfulness
Similarity	**0.838**					
Relevance	0.399	**0.818**				
Overload	0.434	0.292	**0.815**			
Fatigue	0.432	0.303	0.429	**0.936**		
Avoidance	0.410	0.258	0.257	0.372	**0.852**	
Mindfulness	−0.406	−0.384	−0.336	−0.303	−0.312	**0.902**

Next, the model constructed in this article underwent a fit test. Through PLS Algorithm analysis, the R^2^ values of all latent variables were greater than 0.1, indicating that the model’s predictive accuracy is satisfactory (Hair et al., 2017). Subsequent Blindfolding analysis showed that all variables’ Stone-Geisser *Q*^2^ values were greater than 0, suggesting that the research model could effectively predict the relationships between variables (Dijkstra and Henseler, 2015). Additionally, the SRMR value was 0.075, less than 0.08, the RMS Theta value was 0.106, and the NFI value was 0.966, greater than 0.9 (Henseler et al., 2014); overall, the fit indices of the research model passed the test.

### Research hypothesis testing

To test the research hypotheses, a bootstrap resampling with a sample size of 5,000 was performed on the data at a 5% significance level. The test results are shown in Table 4.

**Table 4:** Hypothesis testing results.

Direct effect	β	T	p
H1a: Similarity→Fatigue	0.246	3.254	0.001**
H1b: Relevance→Fatigue	0.097	1.390	0.165
H1c: Overload→Fatigue	0.269	3.908	0.000***
H2: Fatigue→Avoidance	0.307	5.278	0.000***
H3a: Mindfulness→Fatigue	−0.076	−0.965	0.335

Indirect effect	Effect value	*T*	95% CI	*P*
H2a: Similarity→Fatigue→Avoidance	0.075	2.762	[0.025, 0.134]	0.000***
H2b: Relevance→Fatigue→Avoidance	0.030	1.264	[−0.007, 0.086]	0.206
H2c: Overload→Fatigue→Avoidance	0.082	3.009	[0.036, 0.142]	0.003**

Moderating effect	Effect value	*T*	*p*
H3b: Mindfulness*Fatigue→Avoidance	−0.183	−3.556	0.000***

**p < 0.01; ***p < 0.001.

The analysis results indicated that online health information similarity (β = 0.246, *p* < 0.01) and health information overload (β = 0.269, *p* < 0.001) both have a significant positive impact on health information fatigue among the older adults, thus H1a and H1c are supported. However, there was no significant relationship between health information relevance and health information fatigue (*β* = 0.097, *p* = 0.165 > 0.05), leading to the rejection of H2a. Additionally, health information fatigue significantly influences the older adults health information avoidance behavior (*β* = 0.307, *p* < 0.001), supporting H2. The study also found that health information similarity (95% Boot CI = [0.025, 0.134]) and health information overload (95% Boot CI = [0.036, 0.142]) can lead to the avoidance behavior of health information among the older adults through health information fatigue, validating H2a and H2c. However, health information relevance does not impact older adults health information avoidance through health information fatigue (95% Boot CI = [−0.007, 0.086]), hence H2b is not supported. Specifically, within the mediation pathway from Similarity → Fatigue → Avoidance, older adults individuals’ perceived level of health information similarity positively affects health information fatigue (*β* = 0.246, *p* < 0.01), which in turn increases health information avoidance behavior (*β* = 0.307, *p* < 0.001). In contrast, on the pathway from Relevance → Fatigue → Avoidance, the level of health information relevance perceived by the older adults does not trigger health information fatigue (*β* = 0.097, *p* = 0.165 > 0.05). Even though there is a positive correlation between health information fatigue and health information avoidance behavior (*β* = 0.307, *p* < 0.001), this mediation pathway is ineffective. On the pathway from Overload → Fatigue → Avoidance, the level of health information overload perceived by older adults individuals also positively impacts health information fatigue (*β* = 0.269, *p* < 0.001), which likewise enhances health information avoidance behavior (*β* = 0.307, *p* < 0.001).

**Figure 2:**
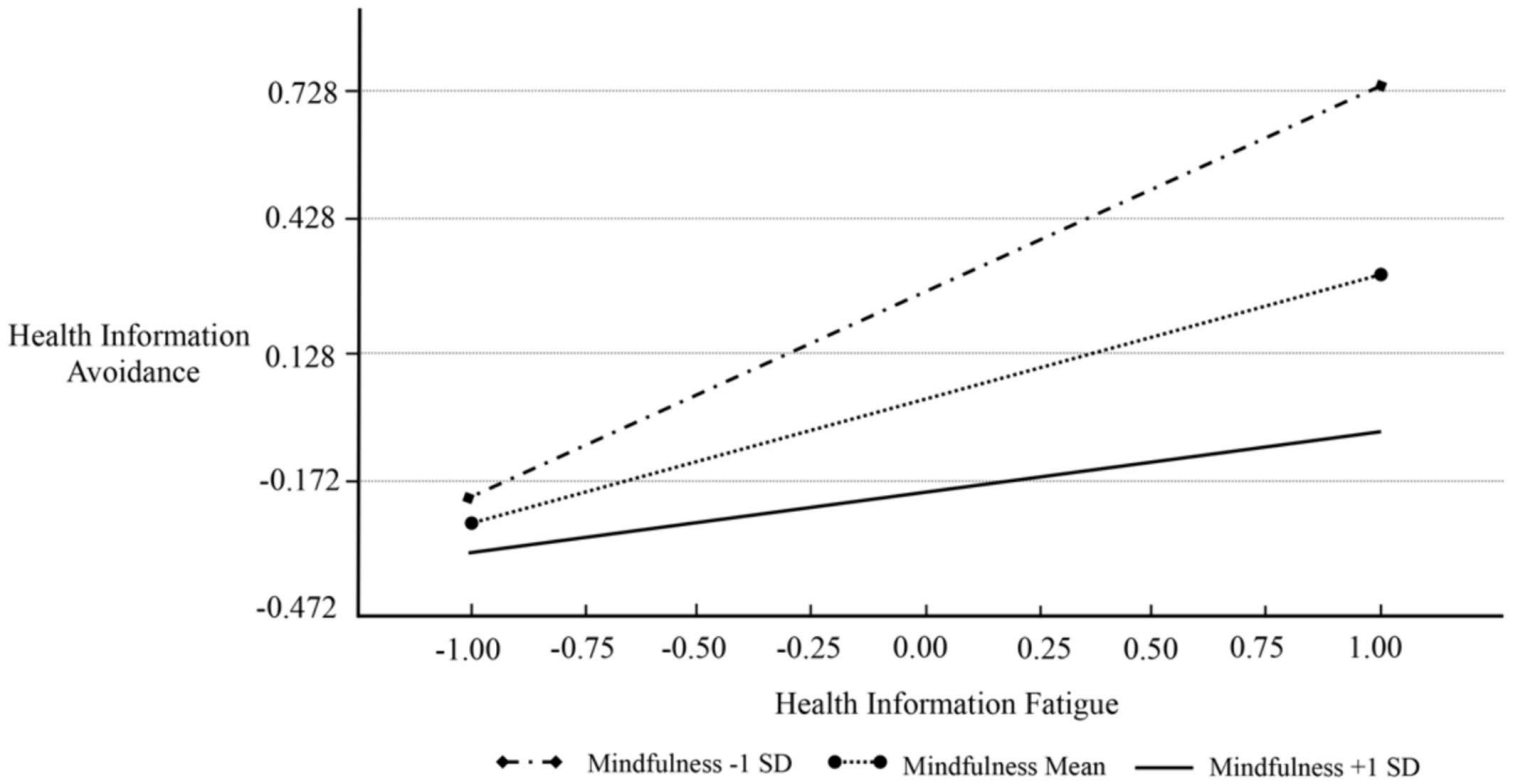
The simple slope test.

Further testing of H3a and H3b was conducted using SPSS Process Model 4 and Model 1, respectively, with Bootstrap resampling repeated 5,000 times. The analysis showed no significant relationship between trait mindfulness and the sensation of health information fatigue (*β* = −0.076, *p* = 0.335 > 0.05), rejecting H3a. Yet, trait mindfulness could negatively moderate (inhibit) the health information avoidance behavior caused by health information fatigue among the older adults (*β* = −0.183, 95% Boot CI = [−0.283, −0.083]), supporting H3b. A simple slope graph illustrating this is shown in Figure 2.

## Discussion and conclusion

Current study aims to investigate the impact mechanism of the health information echo chamber on the avoidance behavior of health information among the older adults and to verify the intervention effect of trait mindfulness on health information avoidance behavior. Based on the Stress-Coping Theory and the SSO framework, the study constructed the impact of information characteristics within the digital environment’s echo chamber on the older adults health information avoidance. Through a cross-sectional survey of 236 older adults individuals, it was found that health information similarity and health information overload within the echo chamber could trigger a sense of fatigue toward health information among the older adults, which in turn exacerbates their avoidance of health information. Additionally, the study verified the moderating factor of trait mindfulness, finding that although trait mindfulness cannot directly reduce the older adults sensation of health information fatigue, it can effectively inhibit the health information avoidance behavior triggered by such fatigue.

### The echo chamber increases health information avoidance among the older adults

In the digital age, the method of distributing health information is heavily influenced by algorithms designed to maximize user engagement. These algorithms often create echo chambers, narrowing the scope of information accessible to users. This effect is particularly pronounced in health communication, where accurate and diverse information is crucial. This study has revealed that such echo chambers significantly exacerbate the tendency of the older adults to avoid health information, which can lead to inadequate health decisions, a failure to recognize health risks timely, or ineffective use of medical resources and services (Terren and Borge-Bravo, 2021). This avoidance is often due to a self-protective reaction against the fatigue caused by excessive similarity and overload of information (Wollebæk et al., 2019), rather than the relevance of the information itself, a finding that contradicts previous research (Jennings et al., 2021). From a practical standpoint, this research underscores the necessity for health communicators—be it companies or media outlets—to develop strategies that escape the limitations imposed by algorithms and effectively reach older audiences. These strategies should ensure that health information is diverse, accessible, and manageable, thus helping to dismantle harmful echo chambers by promoting varied sources and perspectives. This may be due to the fact that highly relevant information is often considered to have greater informational value, which can stimulate older adults users’ cognitive engagement and emotional interest, thereby reducing the psychological fatigue experienced during the information processing (Zimmer et al., 2010). Firstly, information with high relevance can directly address the health concerns and needs of the older adults, such information, often being the solutions or answers they seek, is likely to be regarded as valuable and meaningful. When the information matches individuals’ immediate needs and interests, it not only becomes easier for them to understand, but also enhances their perception of its personal relevance, thus increasing its appeal and reducing the likelihood of it being perceived as a burden. Furthermore, for older adults individuals, highly relevant health information can serve as an incentive, encouraging them to actively engage in managing their health. This active participation can translate into better health behaviors and decisions, providing positive psychological feedback and enhancing their motivation to process and accept new information. Therefore, in the digital information echo chamber, highly relevant information does not directly lead to information fatigue among the older adults, nor does it significantly affect avoidance behavior. When information is considered useful and essential, it effectively promotes the individual’s acceptance and application of health information, thereby alleviating fatigue (Afful-Dadzie et al., 2023). This consideration should be taken into account when designing health information dissemination strategies targeted at the older adults. We also call for future research to explore this further.

Additionally, the introduction of trait mindfulness in health education for the older adults could be a powerful tool to mitigate the avoidance of health information. By cultivating a mindful approach to processing health content, the older adults might manage their information consumption more effectively, avoiding the overwhelm that leads to avoidance. In summary, this study not only deepens our theoretical understanding of health information behavior in the digital age but also provides practical insights for improving health communication strategies. It highlights the critical need for tailored approaches that consider the unique information processing tendencies of the older adults, ensuring they remain informed and engaged in managing their health (Mak et al., 2019).

### Trait mindfulness as an effective moderator in curbing health information avoidance induced by fatigue

This study significantly advances our understanding of how trait mindfulness acts as a moderating factor in health information behavior, particularly among the older adults. It demonstrates that mindfulness can help mitigate the tendency to avoid health information, a behavior often triggered by the fatigue associated with excessive, redundant, or overwhelming health content. Mindfulness encourages older adults individuals to be fully present and non-judgmental about their thoughts, emotions, and bodily sensations concerning their health, fostering a deeper understanding and acceptance of their condition. This heightened awareness helps reduce mental burdens and anxiety, improving their psychological and emotional well-being significantly. Older adults individuals with higher levels of trait mindfulness exhibit a greater capability to engage with available health information critically and openly, even when it appears vast or repetitive. This mindful approach allows them to filter and prioritize health information more effectively, reducing the likelihood of avoidance due to fatigue. Moreover, the practice of mindfulness enhances their overall happiness and self-efficacy in daily life, empowering them to make more informed and beneficial health decisions.

The implications of these findings extend beyond individual health behaviors to influence public health strategies and health communication practices. Health communicators, including companies and media outlets, need to recognize the value of promoting mindfulness as a tool for managing health information consumption. By incorporating mindfulness training into health education programs and outreach initiatives, these entities can help the older adults navigate the complexities of modern health information environments more successfully. In practice, promoting mindfulness could involve creating content that not only informs but also encourages reflective engagement with health information. Media campaigns and health information materials could include mindfulness exercises or prompts that guide the older adults to reflect on how the information relates to their personal health contexts. Such strategies not only enhance the direct impact of health communications but also align with broader public health goals of improving the health literacy and autonomy of older adults populations. In conclusion, the integration of mindfulness into health education and communication strategies represents a promising approach to counter the negative effects of information overload and echo chambers in digital health dissemination. By doing so, health communicators can significantly improve the effectiveness of their messaging and support healthier, more informed decision-making among the older adults.

### Implications

Current study investigates the impact of the health information echo chamber on the older adults health information avoidance behavior and examines the potential of trait mindfulness as an effective intervention. It provides insights into the behavior of the older adults regarding health information in the digital era from the perspective of information dissemination. The theoretical contributions are threefold: first, expanding the health information echo chamber research. This study applies the concept of the echo chamber effect to the health information domain, particularly focusing on the older adults population. It unveils how this phenomenon influences their acceptance and processing of health information, thereby broadening the research horizons of echo chamber effects across various fields. Second, enriching understanding of health information avoidance among the older adults. By constructing a pathway that explains the older adults avoidance of health information, this work illuminates the psychological mechanisms underlying this behavior. This offers new theoretical insights for deciphering the older adults behavioral patterns when faced with health information. Third, demonstrating the effectiveness of trait mindfulness. The study validates trait mindfulness as a regulatory factor against health information avoidance, expanding the applicability of mindfulness research. It suggests new theoretical approaches and frameworks for helping the older adults navigate out of health information echo chambers and enhance the effectiveness of health information dissemination.

The practical implications include: first, improving the health information behavior pattern of the older adults by identifying and understanding the key factors affecting the older adults health information processing, relevant institutions and individuals can design more effective health information dissemination strategies that align with the needs and preferences of the older adults. Second, optimizing the digital health living environment for the older adults, the findings of this article can provide references for governments and public health institutions to more accurately target the needs of the older adults population when formulating relevant policies and intervention measures, promoting the development of an age-friendly society. Third, it offers a new perspective for public health practice and digital health education, emphasizing the importance of integrating trait mindfulness training into health information dissemination and education strategies. By designing and implementing mindfulness training programs targeted at the older adults, it can help them better handle health information, avoid information fatigue and avoidance behavior, while enhancing their health awareness and self-care ability.

### Limitations and future directions

While this study contributes to both theory and practice, it has limitations that offer avenues for future research: first, the study is confined to the perspective of information dissemination within the health information echo chamber, somewhat neglecting psychological and social factors such as individuals’ health beliefs, the level of social support, and technology acceptance. Second, although the study validates the effective intervention of trait mindfulness on the health information avoidance behavior of the older adults, its underlying mechanisms could be further explored. Future research could incorporate discussions on the older adults health digital literacy, attitudes toward aging, and self-efficacy.

Furthermore, this paper also anticipates directions for future research: first, it suggests a deeper exploration and optimization of intervention measures, including different types of intervention methods, implementation strategies, and how to customize intervention plans according to the diverse needs of the older adults. Second, it recommends a more comprehensive consideration and analysis of psychological and social factors affecting the older adults health information processing, such as health beliefs, social support, and individuals’ acceptance of digital technology, to more thoroughly understand and explain the deep-rooted drivers behind the older adults health information avoidance behavior.

## Data Availability

The original contributions presented in the study are included in the article/Supplementary material, further inquiries can be directed to the corresponding author.
